# Familial chilblain lupus due to a novel mutation in *TREX1* associated with Aicardi–Goutie’res syndrome

**DOI:** 10.1186/s12969-020-00423-y

**Published:** 2020-04-15

**Authors:** Cuili Yi, Qiyuan Li, Jihong Xiao

**Affiliations:** 1 Pediatric Rheumatology Unit, Pediatric Department, The First Affilated Hospital of Xiamen University, No. 55 Zhenhai Road, Xiamen, Fujian China; 2 Genokon Medical Laboratory, Xiamen, China

**Keywords:** Familial chilblain lupus, *TREX1*, Mutation, Chinese, Aicardi-Goutières syndrome, Systemic lupus erythematosus

## Abstract

**Background:**

Familial chilblain lupus (FCL) is a rare, chronic form of cutaneous lupus erythematosus, which is characterized by painful bluish-red inflammatory cutaneous lesions in acral locations. Mutations in *TREX1*, *SAMHD1* and *STING* have been described in FCL patients. Less than 10 *TREX1* mutation positive FCL families have been described in the literature.

**Case presentation:**

Genetic study was performed in a large, nonconsanguineous Chinese family with 13 members over 4 generations affected by chilblain lupus. Whole exome sequencing was performed for the index patient. Significant variant detection was subsequently validated by resequencing using Sanger sequencing in the index patient and other family members. A novel pathogenic mutation *TREX1* p.Asp18His was iditified in the index patient. The mutation was present in affected individuals and was absent in non-affected individuals in the familiy.

**Conclusions:**

We present a four-generation Chinese family with FCL caused by a novel heterozygous mutation *TREX1* p.Asp18His, which had been reported in a patient with Aicardi–Goutie’res syndrome. This is the first reported Chinese family with FCL based on mutation in *TREX1*.

## Background

Chilblain lupus erythematosus (CHLE) is a rare, chronic form of cutaneous lupus erythematosus, characterized by painful bluish-red inflammatory cutaneous lesions in acral locations such as fingers, toes, nose, cheeks, and ears, and tend to ulcerate [1]. Cutaneous lesions are precipitated by cold and wet exposure and usually improve during summer. Sporadic CHLE usually affects middle-aged females, whilst familial chilblain lupus (FCL) manifests in early childhood, which was first described by Lee-Kirsch MA. et al. [2] in 2006. FCL is a monogenic form of cutaneous lupus erythematosus, and mostly inherited in an autosomal-dominant trait. Mutations in Three Prime Repair Exonuclease 1 (*TREX1*) [2–11], *SAMHD1* [12] and *STING* [13] have been described in FCL patients. Less than 10 *TREX1* mutation positive FCL families have been described in the literature [2–11]. Here, we report a novel *TREX1* mutation in a Chinese FCL family by whole exome sequencing. This is the first reported Chinese family with FCL based on mutation in *TREX1*.

## Case presentation

**Fig. 1:**
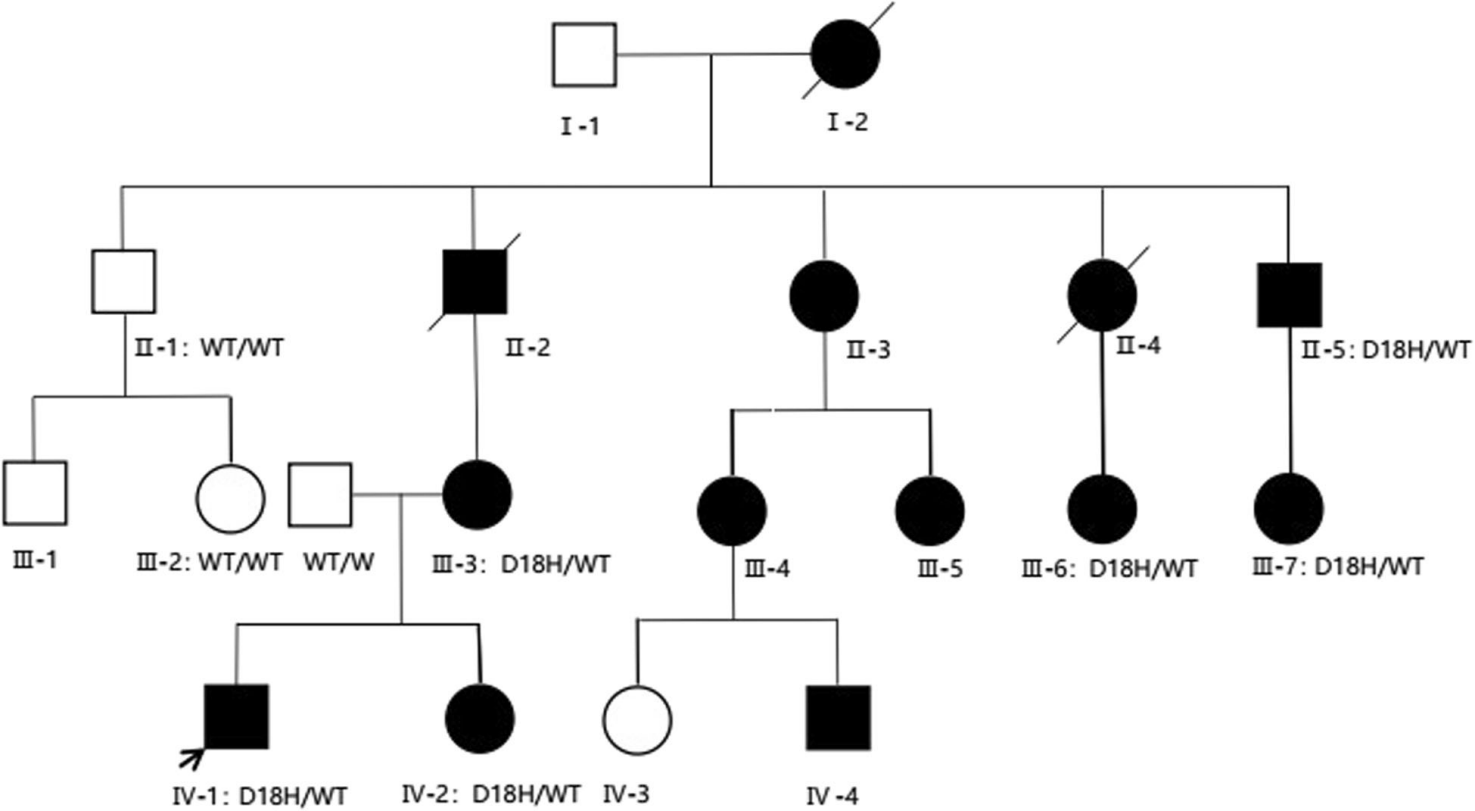
Pedigree of the family with dominant Familial chilblain lupus erythematosus. The arrow shows the index patient (IV-1). Black squares and circles indicate affected males and females; open squares and circles indicate unaffected males and females

**Fig. 2:**
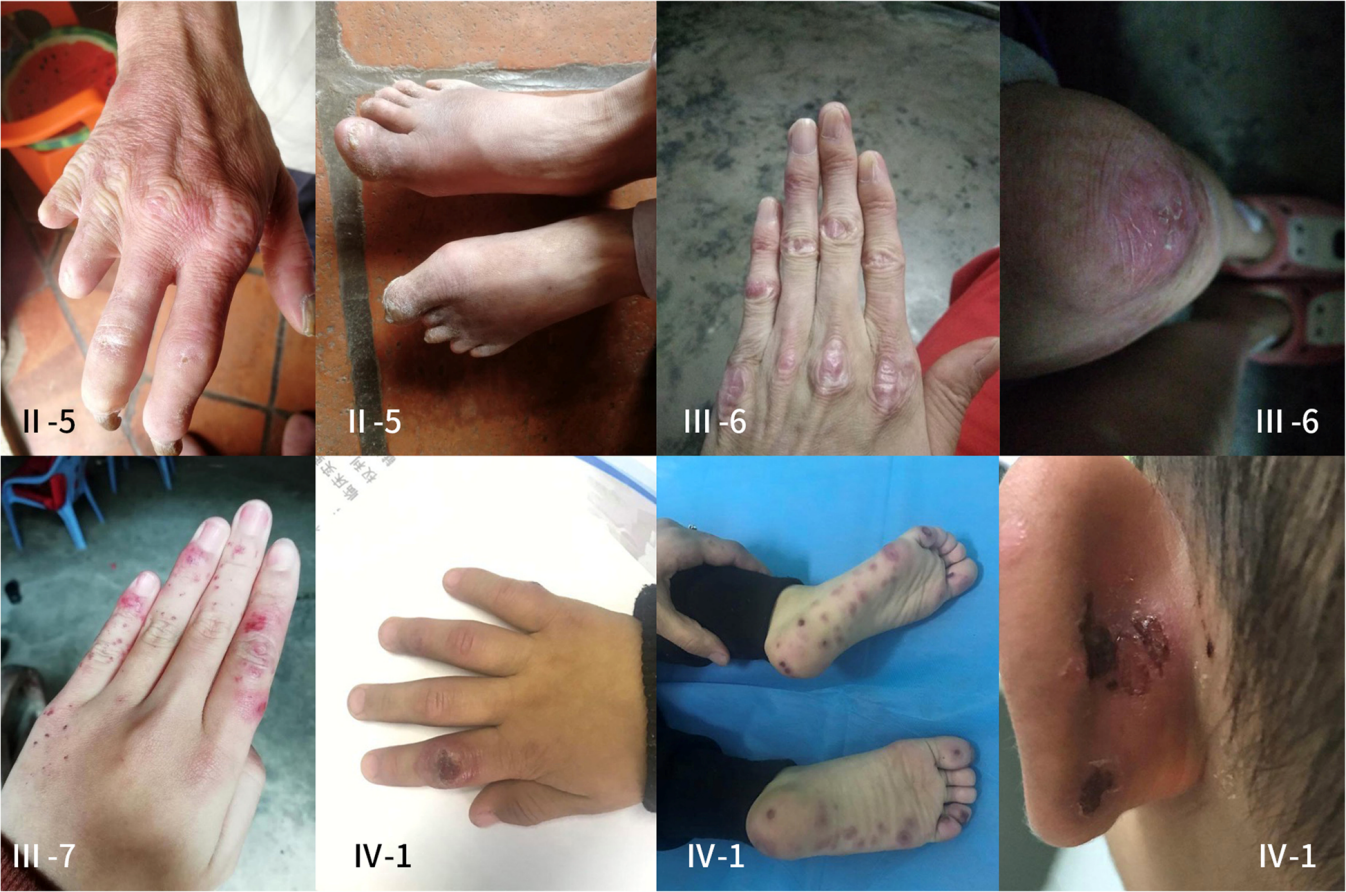
Chilblain lesions on skin of the patients. Skin features observed in the affected families(II-5, III-6, III-7, IV-1). Previous ulcerations have led to a loss of the distal interphalangeal joints in patient II-5

In this study, we describe a large, nonconsanguineous Chinese family with 13 members over 4 generations affected by chilblain lupus (Fig. 1**,** Table 1). All affected individuals showed painful bluish-red papular, or nodular lesions, or even ulcerations of the skin in acral locations including fingers, toes, ears, and nose since early childhood, which became significantly worse in the winter months (Fig. 2). Patients II-3, III-3, III-4, III-5 and III-7 showed great improvements of cutaneous lupus lesions as they grew older, having a few skin lesions only in cold weather now. The condition of Patient III-6 and Patient II-5 did not improve as they aged, having severe skin lesions especially in cold weather now. Patient II-5 even had destruction of the distal interphalangeal joints because of the ulcerations. Except for arthritis in patients III-3, III-6, IV-1, IV-2, there was no history of associated disease of any internal organ (including the central nervous system), immune deficiency, or malignancy in this family.

**Table 1:** Clinical manifestations of 13 affected individuals in the FCL family

ID	Sex	Age	Age of onset (year)	Skin lesions	System involvemenets	Mutation status
I-2	F	Deceased (Unknow reason)	NA	+	NA	NA
II-2	M	Deceased (Vital myocarditis)	NA	+	NA	NA
II-3	F	54	Early childhood	+	N	NA
II-4	F	Deceased (Suicide)	NA	+	NA	NA
II-5	M	47	Early childhood	+	N	+
III-3	F	27	Early childhood	+	Arthritis	+
III-4	F	27	Early childhood	+	N	NA
III-5	F	25	Early childhood	+	N	NA
III-6	F	24	Early childhood	+	Arthritis	+
III-7	F	15	Early childhood	+	N	+
IV-1	M	3.8	0.5	+	Arthritis	+
IV-2	F	1.6	0.6	+	Arthritis	+
IV-4	M	3.9	0.6	+	N	NA

(+) positive; *N* no, *NA* not available

More data was available from three affected individuals who had been hospitalized.

### Patient IV-1

The index patient (IV-1), a 3.8-year-old boy, was born at 39 weeks after an uncomplicated pregnancy to unrelated parents. His birth weight was 3100 g and no congenital infections were documented. He has developed chilblains on his fingers, toes and ears since the first winter when he was 6 months old. He also reported recurrent arthritis of the knees and hip joints since 2 years old. Physical examination was normal except for skin findings such as crusty wounds, hyperemic ulcers on acral surfaces and swelling of the knees. Except for mildly anemia, laboratory test findings were unremarkable, including liver and kidney function tests, urinalysis, erythrocyte sedimentation rate, C reactive protein, rheumatic factor, anticardiolipin antibodies, complement levels, as well as antibodies of extractable nuclear antigens, double-stranded DNA, and cyclic citrullinated peptide. There was no evidence for hypergammaglobulinemia, cold agglutinins, viral or bacterial infection. His cranial CT scan was normal, brain MRI disclosed an abnormal signal in bilateral occipital white matter, suggesting the possibility of poor myelination. MRI of right knee revealed synovitis with effusion. No abnormalities were found in ophthalmological examination. Skin biopsy was not performed.

### Patient IV-2

Patient IV-2 is the sister of the index patient, who was 1.6 years old. She has got chilblains on her fingers and toes since her first winter just like what her brother has. She got the swelling of right knee at 1.2 years old, regressing a few days later. Physical examination showed painful bluish-red inflammatory cutaneous lesions in fingers and toes. Laboratory investigations were unremarkable. Her cranial CT scan was normal. MRI of right knee indicated arthritis.

### Patient III-6

**Fig. 3:**
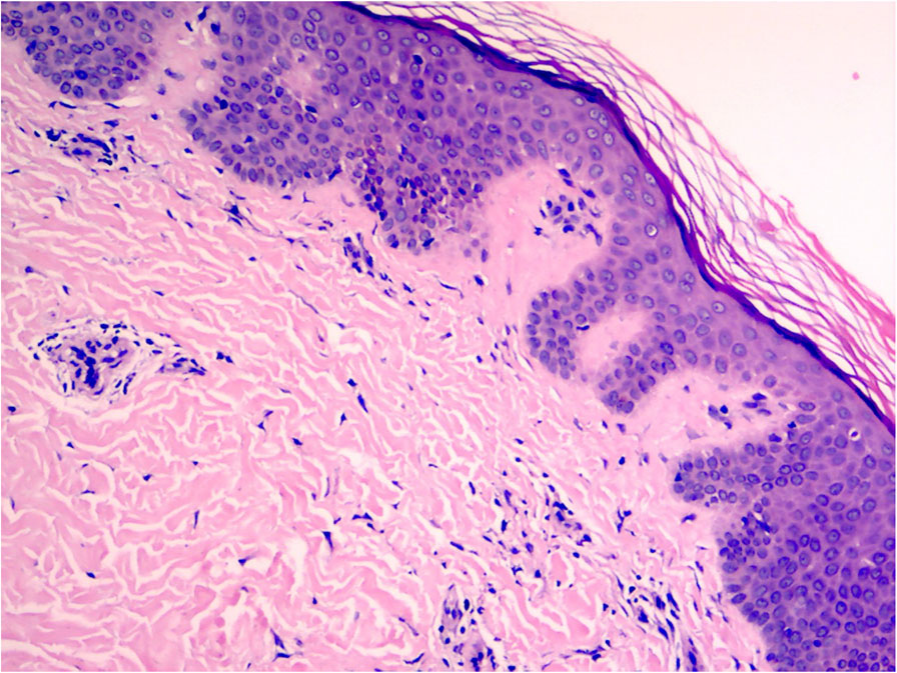
Histology of lesional skin biopsy from Patient III-6

Patient III-6 was an aunt of the index patient, who was in her 20s. She has reported chilblains on her fingers, toes and knees, and arthralgia of the knees since early childhood. Laboratory investigations were unremarkable, except for slightly elevated of erythrocyte-sedimentation rate, serum IgA and IgG. Knees MRI revealed arthritis. Histologic examination of lesional skin from the knee showed lymphocytes, neutrophils and eosinophils dermal inflammatory infiltrate, and focal dermal interstitial edema with cystic degeneration(Fig. 3), which was consistent with lupus erythematosus.

In order to identify the genetic etiology of the disease in this family, whole exome sequencing (WES) (Additional file 1) was performed for the index patient. Significant variant detected was subsequently validated by resequencing using Sanger sequencing in the index patient and other family members, including II-1, II-5, III-2, III-3, III-6, III-7, IV-2 and the index patient’s father. The participants in this study gave written informed consent. This study was approved by the ethical committee of The First Affilated Hospital of Xiamen University.

**Fig. 4:**
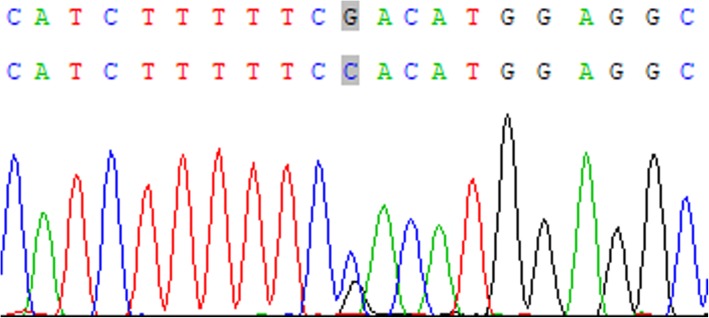
Sequence data of *TREX1* gene in the index patient. The nucleotide exchange is marked by an arrow, with two peaks representing G and C at location 52

Acrroding to “Mayo Clinic Diagnostic Criteria” [1], all patients in this family can be diagnosed as FCL. WES revealed a heterozygous novel missense mutation c.52 G > C in *TREX1* gene leading to a Aspartate to Histidine substitution (p.Asp18His) in the index patient, which was validated by Sanger sequencing (Fig. 4). The mutation was presented in affected individuals II-5, III-3, III-6, III-7, and IV-2. Patient III-3 was the index patient’s mother. The mutation was absent in non-affected individuals II-1, III-2, and the index patient’s father.

## Discussions

TREX1 is a 314 amino acid protein encoded by gene *TREX1*, which is located on chromosome 3p21. It represents the major DNA-specific 3-prime-to-5-prime exonuclease activity measured in mammalian cells. It is anchored in the outer nuclear membrane that degrading short DNA metabolites derived from the nucleus leak into the cytosol [1, 4, 14]. In TREX1-deficient cells, self-DNA accumulates in the cytosol and leads to inappropriate activation of chronic type I interferons, which can break immune tolerance and promote autoimmunity or autoinflammatory diseases [4, 14].

Nine *TREX1* mutation-positive FCL families have been described in the literature [2–11] (Table 2). Among the nine families, five of them had a mutation resulting in a Aspartate to Asparagine substitution at the acid residue 18 (p.Asp18Asn). It shows aspartate 18 is a hot-spot mutation. In contrast, in this study, we found a heterozygous mutation resulting in a Aspartate to Histidine substitution at the acid residue 18, which has not been reported in FCL patients before. Several lines of evidence indicated that the mutation *TREX1* p.Asp18His was pathogenic. First, the acid residue 18 constitutes a highly conserved Mg 2+ − coordinating aspartate residue within the catalytic centre of the dimeric TREX1 enzyme [4], suggesting that it could affect enzymatic function. Second, the mutation was a rare variant not found in 1000 genome, ExAC, gnomAD. Third, the mutation was predicted to be a disease-causing mutation by several different computational prediction methods, including SIFT, Polyphen, Mutation Taster, and PROVEAN. Fourth, this mutation completely cosegregated with affected family members and was absent in non-affected family members. Finally, the clinical course of our patients was similar to those observed in previously reported FCL patients with *TREX1* mutation [2–11] (Table 2).

**Table 2:** Summary of families previously reported with Familial Chilblain Lupus based on mutation in *TREX1*

Ethnic	No. of patients	Age of onset	Modes of inheritance	Mutation	Clinical manifestations (No. of patients)	Immunologic findings	Skin biopsy findings (No. of patients)	References
Turkey	2	2.5	AR	p.Arg114Cys	Skin lessions (2) Cerebral vasculopathy (1)	Positive: free protein C was mildly reduced Negative: C3,ANA, dsDNA, CryoG, CryoF, anticardiolipin antibodies, Antithrombin III, protein S and homocysteine levels	NA	3
Germany	18	2.3	AD	p.Asp18Asn	Skin lessions (18) Arthritis (4)	Positive: ANA Negative: RF, Cold agg,CryoG, CryoF, anticardiolipin antibodies	Consistent with LE(3)	2, 4
Japan	5	early childhood	AD	p.Asp18Asn	Skin lessions (5) Cerebral vasculopathy (1) subarachnoid hemorrhage (1)	Positive: ANA, an increased interleukin-6 of cerebrospinal fluid Negative: anticardiolipin antibodies,CryoG, Antithrombin III, protein, homocysteine levels and free protein C was mildly reduced	Small vessel angitis(1)	5
Germany	4	childhood	AD	p.His195Gln	Skin lessions (4) Arthritis (3) thrombocytopenia (3) lymphocytopenia (3)	Positive: ANA 1:160 Negative: C3, RF, CCP	Consistent with LE(2)	6
Bangladeshi	4	3	AD	c.375dupT	Skin lessions (3) Arthritis (2)	Positive: ANA1:1000 Negative:RF,C3,ENA, CryoG, CryoF, anticardiolipin antibodies,	NA	7
Japan	10	Early Childhood	AD	p.Asp18Asn	Skin lessions (10)	NA	NA	8
Germany	4	Childhood	AD	p.Asp18Asn	Skin lessions (4) photosensitive rash (1)	Positive: ANA 1:80 Negative: C3, CryoG,	Consistent with LE(1)	9
Japan	6	early childhood	AD	p.Pro132Ala	Skin lessions (6)	Positive: ANA 1:80 Negative: C3, dsDNA	Consistent with LE(1)	10
Germany	3	childhood	AD	p.Asp18Asn	Skin lessions (3) Arthritis (1) Leukopenia, animia, thrombocytopenia (1)	Positive: ANA 1:160, elevated of immuno-globulin G Negative: ENA, anticardiolipin antibodies,CryoG,	Consistent with LE(1)	11
China	13	early childhood	AD	p.Asp18His	Skin lessions (13) Arthritis (4)	Positive: Negative: CCP, RF, ANA, ENA, C3,anticardiolipin antibodie, cold agg,	Consistent with LE(1)	Present case

*ANA* anti-nuclear antibody, *Cold agg* cold agglutinin, *CryoG* cryoglobulin, *CryoF* cryofibrinogen, *dsDNA* double-stranded DNA, *RF* rheumatic Factor, *C3* complement 3, *LE* lupus erythematosus, *ENA* antibodies of extractable nuclear antigens, *CCP* cyclic citrullinated peptide, *NA* not analyzed, *AR* autosomal recessive, *AD* autosomal dominant

In addition, the heterozygous *TREX1* mutation (c.52G > C; p.Asp18His) has been reported in a patient with Aicardi-Goutières syndrome (AGS) [9]. AGS is a rare syndrome characterized by calcification, diffuse demyelination, and variable degree of brain atrophy caused by inherited defects in nucleic acid metabolism [15]. About 24% of AGS patients have mutations in *TREX1* [16, 17]. And chilblain-like lesions are observed in 36.7% of *TREX1* AGS patients [15]. Haaxma CA et al. [18] reported a de novo heterozygous p.Asp18Asn mutation in *TREX1* in an AGS patient, which was the most frequent mutation in FCL patients. Abe J et al. [8] reported a case of AGS and FCL in a three-generation family with chilblains caused by the same heterozygous *TREX1* p. Asp18Asn mutation. Apart from AGS and FCL, mutations in *TREX1* are also responsible for systemic lupus erythematosus (SLE). SLE is a heterogeneous multisystem autoimmune disease, characterized by a variety of clinical manifestations and a wide profile of autoantibodies. An upregulation of type I interferon signaling has been reported in some SLE patients [19]. About 2% SLE patients have mutations in *TREX1* [15]. Namjou et al. [20] reported a mutation *TREX1* p.Arg114His in an SLE patient, which was the most frequently *TREX1* mutation in AGS patients. There are some clinical, genetic, and basic science considerations that underline a possible overlap between AGS, FCL and SLE. But the exact molecular mechanisms and the different modes of inheritance remain to be clarified.

Apart from cutaneous lesions, signs of systemic involvement have been observed in FCL patients, including arthralgia, cerebral thrombosis and hematologic system involvement including apenia, leukopenia, thrombocytopenia, and some patients have elevated of antinuclear antibodies titer [2–11] (Table 2). Millard LG et al. [21] reported that up to 18% of affected sporadic CHLE individuals progressed to SLE after a long time of follow up, which was not found in FCL patients. The high prevalence of systemic clinical manifestations may suggest that TREX1-associated FCL may be a systemic disease with prominent cutaneous involvement.

The expression of the phenotype may vary among the members of an individual FCL family with *TREX1* mutation. In the family described in this study, patients II-3, III-3, III-4, III-5 and III-7 had cold-induced infiltrates and ulcerations in childhood that declined in severity as they aged, whereas patient II-5 had destruction of the distal interphalangeal joints because of the ulcerations. Arthralgia was presented in patients IV-1, III-3, IV-2 and III-6, but not in other affected patients in our study. *TREX1* mutation FCL patients may have variable penetrance, and the same mutation can cause an exclusive skin phenotype, or a neurological phenotype, or a hematologic system involvement, even in the same family [2–11] (Table 2). Gillian Rice et al. [7] reported one individual in an FCL family was unaffected on clinical examination but carried the same molecular changes observed in her affected siblings. Modifier genes and their epistatic interactions, epigenetic or environmental factors may also play a role in the result of incomplete penetrance, though more cases are needed for a better understanding about these effects.

Patient IV-1 and patient IV-2 have undergone treatment with JAK inhibitor tofacitinib for 2 months. Their symptoms of arthritis had a complete remission and their skin lesions also had a significantly improvement. Patient III-6 was considered to be treated with tofacitinib recently. The exact effect needs a longer follow up of patients IV-1, IV-2 and III-6, especially in winter. The other patients did not have any treatment.

In conclusion, we presented a four-generation Chinese family with FCL caused by a novel heterozygous mutation *TREX1* p.Asp18His, which had been reported in a patient with AGS. This is the first reported Chinese family with FCL based on mutation in *TREX1*.

## Supplementary information

**Additional file 1.**

## Data Availability

The datasets supporting the results of this article are included within the article and its additional file.

## Abbreviations

CHLE: Chilblain lupus erythematosus
FCL: Familial chilblain lupus
WES: Whole exome sequencing
TREX1: Three prime repair exonuclease 1
AGS: Aicardi-Goutières syndrome
SLE: Systemic lupus erythematosus
