# Supplementary material for: Familial chilblain lupus due to a novel mutation in TREX1 associated with Aicardi–Goutie’res syndrome
Source: Pediatr Rheumatol Online J. 2020 Apr 15;18:32. doi: 10.1186/s12969-020-00423-y (PMC7158086; doi:10.1186/s12969-020-00423-y)
Supplement: Supplementary file 1 — Additional file 1. [file 12969_2020_423_MOESM1_ESM.doc]

**Additional file 1**

**Whole-exome sequencing(WES)**

In accordance with the principle of informed consent, peripheral blood (2 ml) were collected from the proband and some of his family members (Ⅱ-1, Ⅱ-5, Ⅲ-2, Ⅲ-3, Ⅲ-6, Ⅲ-7, Ⅳ-2 and the index patient’s father), maintained in tubes with EDTA anticoagulant. Whole-exome sequencing was performed to identify any underlying pathogenic mutation in the proband. First, the genomic DNA was extracted from the leucocyte in the blood sample using Blood Genome Extraction Kit (Tiangen Biochemical Technology (Beijing) Co., Ltd.) and quantitated by Qubit 3.0 Fluorometer (Thermo Fisher). DNA was then sheared by Bioruptor Pico Sonication System. NEBNext Ultra II DNA Library Prep Kit was used for library preparation and NextSeq 500 Sequencing System (Illumina) was used to perform 150bp pair-end sequencing by Genokon Medical Laboratory, Xiamen, China.

Quality control of the raw sequence data was performed by Trimmomatic and data with low quality was removed. Burrows-Wheeler Alignment tool (BWA) was used to align reads to the reference (GRCh37/hg19). GATK (https://software.broadinstitute.org/gatk/) was used for variant discovery and genotyping and ANNOVAR was used to annotate genetic variants with functional information. Common variants were then filtered out, such as variants of intergenic, intronic, upstream, downstreamor intronic or synonymous variants, and variants with minor allele frequency (MAF)>1% in 1000 genome, ExAC and gnomAD database etc. Besides, many methods, including SIFT，PROVEAN，MutationTaster, Polyphen, etc, were used to predict the deleterious affect on the function of proteins of each variant.

**Sanger sequencing**

Sanger sequencing was performed to validate the mutation in the proband and other family members.
